# Impact of insecticide-treated bednets and indoor residual spraying in controlling populations of *Phlebotomus duboscqi*, the vector of *Leishmania major* in Central Mali

**DOI:** 10.1186/s13071-018-2909-2

**Published:** 2018-06-14

**Authors:** Cheick Amadou Coulibaly, Bourama Traore, Adama Dicko, Sibiry Samake, Ibrahim Sissoko, Jennifer M. Anderson, Jesus Valenzuela, Sekou F. Traore, Ousmane Faye, Shaden Kamhawi, Fabiano Oliveira, Seydou Doumbia

**Affiliations:** Leishmaniasis Unit, Department of Entomology of International Center of Excellence in Research (ICER-Mali), Faculty of Medicine and Odontostomatology, University of Science, Techniques and Technologies of Bamako (USTTB), BP 1805 Bamako, Mali

**Keywords:** Cutaneous leishmaniasis, Indoor residual spraying, Sand fly, LST

## Abstract

**Background:**

Cutaneous leishmaniasis (CL) is an endemic neglected tropical disease prevalent in several areas where seasonal malaria transmission is active. We assessed the effect of indoor residual spraying (IRS) and the mass distribution of long-lasting insecticide-treated bednets (LLINs) for malaria control on sand fly population diversity and abundance, and its impact on the risk of *Leishmania* transmission in the district of Baroueli, endemic for CL in Mali.

**Methods:**

Kemena and Sougoula, two villages in the district of Baroueli, were selected for entomology surveys from March to September 2016 to evaluate sand fly species composition and density, and *Leishmania* infection rates in the vector *Phlebotomus duboscqi*. The surveys followed an annual indoor residual spraying and mass distribution of long-lasting insecticide-treated bednets (IRS/LLINs) that began in 2011 for malaria vector control. We also carried out a leishmanin skin test (LST) survey in the two villages to determine the incidence of *Leishmania* infection in humans living in the endemic area.

**Results:**

A total of 2936 sand fly specimens, 1013 males and 1923 females, were collected and identified from the two villages throughout the study period. Fourteen species, 2 belonging to the genus *Phlebotomus* and 12 to the genus *Sergentomyia*, were documented. The genus *Sergentomyia* constituted 91% of collected sand flies *versus* 9% for the genus *Phlebotomus* (*P. duboscqi* and *P. rodhaini*). Of those, *P. duboscqi* was the most abundant, representing 99.6% of the collected *Phlebotomus* species. In both villages, *P. duboscqi* was most abundant during the end of dry season (June). The prevalence of *Leishmania* infection in individual females of *P. duboscqi* by PCR was 3.5%. After 5 years of the IRS/LLINs, the incidence of *Leishmania* infection in the human population as measured by LST was 4.2%.

**Conclusions:**

Compared to historical data collected from 2005–2008, a considerable reduction was observed in both sand fly density and prevalence of human *Leishmania* infection in the villages of Kemena and Sougoula, Baroueli District, following IRS/LLINs. This suggests that IRS/LLINs used for mosquito control also impacts sand fly vectors reducing the incidence of leishmaniasis.

**Trial registration:**

NCT00344084. Registered: 23 June 2006

**Electronic supplementary material:**

The online version of this article (10.1186/s13071-018-2909-2) contains supplementary material, which is available to authorized users.

## Background

The District of Baroueli is endemic for *Leishmania major* [1]. From 2005–2008, we carried out longitudinal entomological and epidemiological surveys to study the seasonality of *L. major* infection rates in sand fly vectors and assessed the prevalence and incidence of *Leishmania* infection in individuals living in two endemic villages, Kemena and Sougoula, in Baroueli District [1]. We found, that *P. duboscqi* is the major vector of cutaneous leishmaniasis (CL) in Mali representing 99% of collected *Phlebotomus*. The overall infection rate in *P. duboscqi* in the two villages was estimated at 2.7% [2]. In 2006, a LST survey established the prevalence of infection in the inhabitants of the two villages at 31%. The incidence was determined to be 10.0% (18.5% for Kemena and 5.7% for Sougoula) and 9.0% (17.0% for Kemena and 5.7% for Sougoula) in both villages combined for 2007 and 2008, respectively [1].

Malaria vector control programmes are commonly based on the use of long-lasting insecticide-treated nets (LLINs) and indoor residual spraying (IRS) with insecticides against mosquitoes. After the failure of the 1960s eradication campaign of malaria [3], IRS was reintroduced in Mali by the National Malaria Control Programme (NMCP) in 2008 supported by United States President Malaria Initiative (PMI) with the aim of reducing the malaria burden, especially among children under five years and pregnant women [4]. The spray programme started in the districts of Koulikoro and Bla and was extended to Baroueli District in 2011.

The IRS/LLINs programme was initiated in Baroueli with the use of a carbamate class insecticide (bendiocarb) from 2011 to 2013. However, because of the observed relatively short residual half-life of bendiocarb (two months), the NMCP switched to a long-lasting (up to six months) version of an organophosphate (OP) class insecticide (pirimiphos-methyl, actellic CS) in 2014 [4]. It is well known that the use of the IRS against malaria vectors can indirectly affect other indoor resting insects such as sand flies, the vectors of *Leishmania* parasites [5]. Indeed, a significant reduction in visceral leishmaniasis (VL) incidence was observed in the Indian sub-continent following an anti-malarial IRS campaign with dichlorodiphenyltrichloroethane (DDT) [6]. Recently, focal applications of DTT residual formulations and/or pyrethroids demonstrated effectiveness against the VL vectors *Lutzomyia longipalpis* in Latin America and *Phlebotomus argentipes* in India [7, 8]. It has also been reported that IRS reduced the density of sand flies in treated *versus* non-treated sites in Iran [9].

The availability of historical *Leishmania* prevalence and incidence data, and monthly sand fly density data, for the villages of Kemena and Sougoula prior to the initiation of the IRS/LLINs programme provides an opportunity to assess changes in the epidemiology of CL transmission in these two villages as a result of the malaria vector control campaign in Baroueli. Here, we report on a seven-month survey to determine the diversity of sand flies, the seasonal abundance of *P. duboscqi*, and the incidence of *Leishmania* infection as measured by LST in the two villages of Kemena and Sougoula after the introduction to the area of a malaria mosquito control campaign in 2011.

## Methods

### Study sites

The study was carried out in Kemena (12°339'N, 6°339'W) and Sougoula (13°059'N, 6°539'W), in the Baroueli Health District, Region of Segou, Mali. The villages are 5 km apart and share the same topography and climate. Each village has approximately 1000 inhabitants. Both villages are organized into a labyrinth of adjoining compounds within which a single extended family resides in several sleeping, cooking and storage houses. The houses are constructed of clay bricks plastered with mud and straw, and with thatched or metal roofs. Domestic animals, such as goats, sheep, horses, cows, donkey and chickens are kept within the confines of a family compound. There is no electricity or running water in these villages. Most of the land surrounding each village is dedicated for agricultural use. For the purpose of this study, data collected from the two villages were combined and referred to as the study area.

The climate is moderate with three distinct seasons: a cool season (20–34 °C); a dry season from (27–40 °C); and a rainy season (25–35 °C). The total annual rainfall is 82.42 mm. Vegetation is sparse and is characterized by the presence of sporadically placed trees such as shea (*Vitellaria paradoxa*), acacia (*Faidherbia albida*), neem (*Azadirachta indica*) and small bushes [2].

### Indoor residual spraying and long-lasting insecticide-treated nets activities

IRS/LLINS were reintroduced in Mali in 2008 through the support of US President Malaria Initiative (PMI). The programme started with the districts of Koulikoro and Bla and was extended to Baroueli District (were Kemena and Sougoula are located) in 2011 with the use of two insecticides: carbamate (bendiocarb) from July 2011 to July 2013, and an organophosphate (pirimiphos-methyl) from July 2014 to date. This activity has been carried once a year for all houses in the selected villages. Therefore, the study area was sprayed once in July 2016 during our sand fly collections.

### Sand fly collection

Sand fly collections were carried out three times a month from March to September 2016. CDC light traps, sticky traps and mouth aspirators were used inside the rooms (indoor collection), outside the rooms (courtyard) and around the villages of Kemena and Sougoula.

A total of 7 light traps were set-up in five randomly selected compounds per village for three consecutive nights. Five light traps were placed inside the bedrooms for indoor collection, and two were placed outside the bedrooms for outdoor collection, for three consecutive nights per month per village. Light traps were placed at dusk and collected at dawn the next day. Additionally, a total of 20 sticky traps were used, 10 traps placed inside the room (one per room) and 10 placed in the holes of trees (one per tree) around the village for three consecutive nights per month, per village.

For sand fly collection by mouth aspiration (resting collection), three people conducted the collection in rooms from three different compounds. In each room, one person aspirated for 20 min in the morning and 15 min in the afternoon for two consecutive days per month per village. In the field, after capture, live *Phlebotomus duboscqi* flies were used for *Leishmania major* parasite detection. The dead sand flies from light trap and resting collections were stored in 70% ethanol until processed.

### Sand fly species identification

In the laboratory, specimens stored in 70% ethanol were placed onto a glass slide containing the lacto-phenol, a clearing solution (Bioquip product, catalog number 6373A, 2321Gladwick St Rancho Dominguez, CA 90220 USA) for morphological identification. Identification was based on the morphology of the male genitalia and female spermathecae and pharynges using a dichotomous key [10] and a CD ROM titled “Les phlébotomes d'Afrique de l'Ouest: logiciel d'identification et d'enseignement” for sand fly identification from the Institut de Recherche pour le Développement (IRD), Edition 2000 [11].

### Detection of *Leishmania major* in sand flies

#### Microscopic detection of *L. major*

Only live *Phlebotomus* females collected with light traps and aspirators were examined microscopically. After collection, all females were first washed with soapy water and *Phlebotomus* sand flies were separated by their size and identified morphologically. The midgut of each *Phlebotomus* female was then dissected in PBS and inspected for the presence of *L. major* parasite by microscopic observation.

#### Molecular detection of *L. major*

Each *Phlebotomus* female specimen was placed in a 1.5 ml microcentrifuge tube containing 25 μl Tissue Lysis buffer (Qiagen, Hilden, Germany). After an overnight incubation at 4 °C the tissue was macerated using a pestle for 2 min. Total DNA was purified using the QIAamp DNA Mini Kit (Qiagen), the DNA concentration of each extraction was determined using a NanoDrop (Thermo Scientific Inc., Wilmington, DE, USA). *Leishmania* DNA was detected by PCR using forward and reverse primers specific for *Leishmania* spp. (Uni21/Lmj4) as described in [2].

### Assessment of exposure to *Leishmania* parasites in humans

To assess recent exposure to *Leishmania* parasites, a LST survey was carried out in each village on individuals who were LST negative during the preceding LST survey carried out in 2008 [1], as well as children within the age group 1–8 years that were born after 2008. After thorough explanation of the study, adult study participants were asked to sign a consent form. Parents or the guardians were invited to sign a consent form for participating children.

### Leishmanin skin test (LST)

The LST (leishmanin, IRC 1228181375, LOT #127) was supplied by the Pasteur Institute, Tehran, Iran. Each test vial contains 1 ml phosphate buffered saline, 0.1% thimerosal, and 6 × 10^6^ killed *L. major* promastigotes [12, 13]. A dose of 0.1 ml of leishmanin was injected intradermally on the inner surface to the left forearm. The diameter of the induration was measured 48 h to 72 h after the injection. Measurements with a diameter greater than 5 mm were considered positive [12].

### Active and passive case detection

For active case detection, interviews and clinical investigation were conducted at the beginning of the study through house-to-house visits by dermatologists to screen for CL lesions. A physical examination was carried out on household inhabitants to search for lesions (locations inspected include head/neck, trunk, upper extremity, lower extremity). Additionally, the local clinic was visited on every trip to inquire about any reported skin lesions. If a suspected lesion is found, it was photographed, and non-invasive scrapings of the border of the lesions were taken for testing by both PCR and microscopy for *L. major* detection.

### Statistical analysis

The LST data were recorded on a case form (CRF), entered in iDataFax management (Version 2014.1.0), and analyzed using the Statistical Package for the Social Sciences (SPSS, Chicago, IL, USA). Descriptive analyses were used to assess the association between LST and demographic variables. A Fisher’s exact test was used to evaluate statistical significance, with significance set at < 0.05.

## Results

### Sand fly population composition, density and infection rates after 5 years of an IRS/LLINs campaign targeting malaria vectors

From March to September 2016, during cyclic IRS/LLINs, a total of 2936 sand fly specimens (1013 males and 1923 females) were collected and identified from the two villages (Table 1). Fourteen species, 2 belonging to the genus *Phlebotomus* and 12 to the genus *Sergentomyia*, were documented. The genus *Sergentomyia* constituted 91% of collected sand flies *vs* 9% for the genus *Phlebotomus* (*P. duboscqi* and *P. rodhaini*). Of the *Phlebotomus* species, *P. duboscqi* was the most abundant, representing 99.6% of those collected (Table 1). Of the *Sergentomyia* species, *S. schwetzi* represented the majority with 42.8% of collected specimens, while *S. dubia* was the second most abundant at 16.8%.

**Table 1:** Sand fly diversity in the study area, March to September 2016

Genus	Species	Males	Females	Total	Percent^a^
*Phlebotomus*	*P. duboscqi*	132	131	263	8.96
	*P. rodhaini*	0	1	1	0.03
*Sergentomyia*	*S. schwetzi*	439	818	1257	42.81
	*S. antennata*	87	403	490	16.60
	*S. dubia*	208	287	495	16.86
	*S. fallax*	2	6	8	0.27
	*S. buxtoni*	1	7	8	0.27
	*S. clydei*	81	130	211	7.19
	*S. affinix vorax*	4	11	15	0.51
	*S. squamipleuris*	44	94	138	4.70
	*S. africana*	14	29	43	1.46
	*S. darlingi*	1	0	1	0.03
	*S. bedfordi*	0	6	6	0.20
	*S. christophersi*	0	0	0	0
Total		1013	1923	2936	100

^a^Percent of the total number of collected sand flies

**Table 2:** Number of collected sand flies per household per month from in the study area

House	March	April	May	June	July	August	September	Total
K1	4	10	6	3	5	3	0	31
K2	16	9	4	29	34	12	11	115
K3	9	32	43	28	5	15	19	151
K4	4	17	13	57	28	7	28	154
K5	4	13	10	27	11	4	0	69
S1	1	3	9	8	2	0	2	25
S2	4	3	2	20	8	3	9	49
S3	2	6	27	4	0	1	4	44
S4	2	8	9	7	2	3	0	31
S5	9	33	61	23	7	2	24	159
Total	55	134	184	206	102	50	97	828
Mean								82.80
Median								59.00
Minimum								25
Maximum								159
Variance								3123.29
Std. Deviation								55.89

**Fig. 1:**
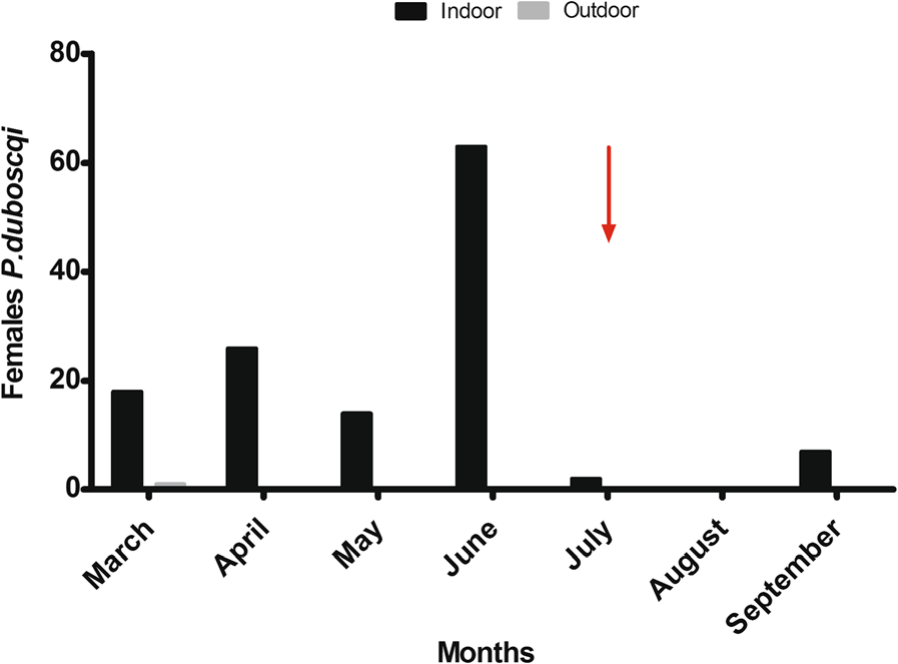
Monthly variation of *P. duboscqi* females collected indoors and outdoors from March to September 2016. Arrow indicates IRS/LLIN conducted for 2016

To assess the impact of IRS/LLINS on CL, we focused on the distribution of the female vector sand fly *P. duboscqi.* Throughout the study period, the majority of *P. duboscqi* sand flies (99.2%, *n* = 130) were collected inside dwellings (Fig. 1). *Phlebotomus duboscqi* showed seasonal fluctuations and was most abundant in June towards the end of the dry season (Fig. 1) and (Additional file 1: Table S1). Importantly, in August, one month after the 2016 annual IRS/LLINs (Fig. 1), no *P. duboscqi* sand flies were collected but *Sergentomyia* were collected in all households except for household S1 (see Table 2).

**Fig. 2:**
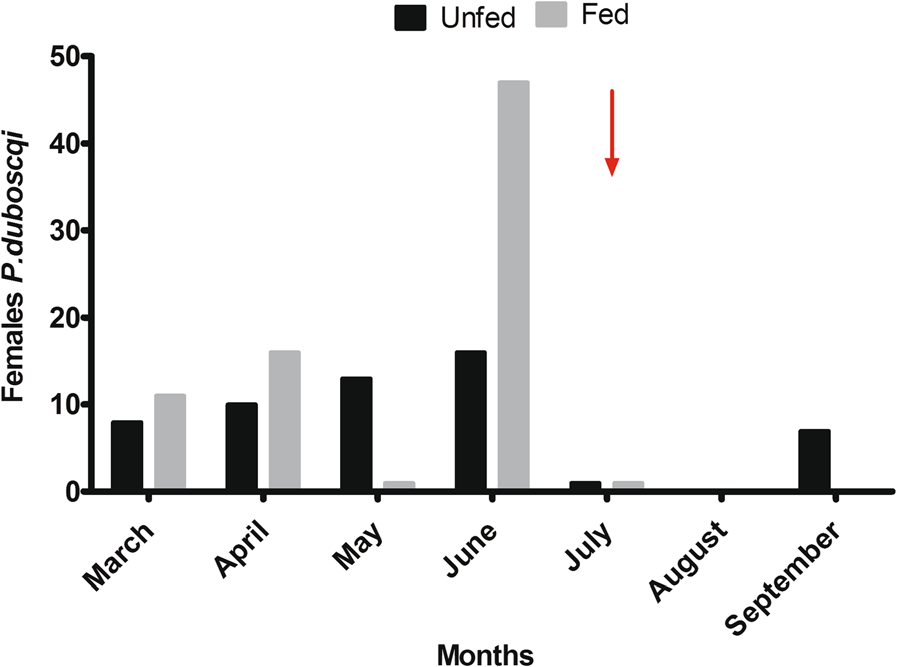
Repartition of female *P. duboscqi* according to the repletion status by month from March to September 2016. Arrow indicates IRS/LLIN conducted for 2016

From March to June 2016, 61% (*n* = 75) of *P. duboscqi* females were blood-fed, from July to September (after IRS/LLINs in July 2016) the repletion status was significantly reduced to 11% (*n* = 1; *P*-value = 0.000001) (Fig. 2).

**Fig. 3:**
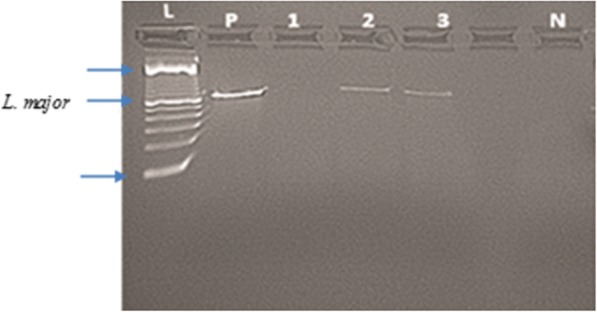
*Leishmania-*specific PCR products from field-collected female *P. duboscqi.* Lane L: 100 bp ladder; Lane P: positive control (DNA from *P. duboscqi* experimentally infected with *L. major*); Lane N: negative control; Lane 1: negative sample; Lanes 2 and 3: positives samples. Predicted band size of the PCR product for *L. major* is 650 bp

Of 86 *P. duboscqi* females processed individually for PCR, 3.5% were found infected (Fig. 3). Microscopic observation of sand fly midguts did not detect parasites (64 *P. duboscqi* females dissected).

### The incidence of exposure to *Leishmania* infection in humans after 5 years of IRS/LLINs campaign

To determine if the five-year IRS/LLINs application had an impact on the exposure of humans residing in the study villages to *Leishmania*, we assessed the incidence of *Leishmania* infection by LST after August 2016 and 5 years of the IRS/LLINs campaign.

Notably, the incidence of *Leishmania* infection in the study area by LST was 4.2% in August 2016. Remarkably, we observed that all the LST positive individuals in this study were 10 years of age or older. No LST positive individuals were found among children aged 1–9 years-old (Table 3). Passive and active detection did not find any CL cases in the study villages during the study period.

**Table 3:** Frequency of *Leishmania* exposure by age group in the study area in August 2016

Age group (years)	No. tested	No. positive	Percent (%)
1–4	274	0	0
5–9	359	0	0
10–14	229	7	3.1
15–19	98	6	6.1
20–24	78	9	11.5
25–29	56	5	8.9
> 30	231	29	12.6
Total	1325	56	4.2

## Discussion

Vector control is an essential strategy for elimination of vector-borne diseases around the world. Among the various options for vector control, IRS/LLINs with appropriate insecticides is a key intervention. It can be used to reduce vector populations and interrupt disease transmission [14].

To our knowledge, this study is the first attempt for evaluation of the effect of IRS/LLINs against sand fly populations in Mali. The impact of malaria vector control on the sand fly fauna was clear. Overall, our results indicated that the IRS/LLINs has had an impact on the sand fly vector density and as a result on the incidence of CL in humans. These two indicators decreased considerably after five years of annual IRS/LLINs application in the study area. The substantial reduction in the density of *Phlebotomus duboscqi*, from 9532 sand flies in 2005 [2] to 2936 in the current study, and the reduction in the incidence of exposure to *Leishmania* infection in humans, from 9.0% in 2008 [1] to 4.2% for the period 2008–2016, is likely attributable to the collateral benefit of the five years of the IRS/LLINs campaign. Notably, we did not observe major changes to the villages during this study (no modification to the houses, no electricity and presence of domestic animals) compared to 2005 [2]. Furthermore, the total rainfall and mean monthly temperature were similar at 82.42 mm, and 30 °C *vs* 78.85 mm and 29 °C in 2005 and 2016, respectively (see Additional file 2: Table S2 for annual average). Interestingly, the presence of *L. major* infection in the sand fly vector at similar proportions, supportive of the presence of an animal reservoir, before and after IRS/LLINs suggests that although CL transmission to humans was ablated by the reduction in the number of vector sand flies after the IRS/LLINs campaign. This effect can be reversed by disruptions in the IRS/LLINs approach, potentially leading to epidemic outbreaks in newly enriched pools of naïve subjects.

It has been demonstrated in Bangladesh, India and Nepal that IRS, but not long-lasting insecticide nets, is effective at reducing *P. argentipes* populations, and that IRS subsequently reduced the number of cases of VL [12]. In Morocco, both IRS with a-cypermethrin and LLINs distribution reduced the incidence of CL [15]. IRS was highly effective, whereas the evidence for LLINs effect was weak and not statistically significant [15].

The sensitivity of *P. duboscqi* to the insecticides used here was remarkable, with a 10-fold reduction in its density from 2605 in 2005 [2] to 263 in the current study. Additionally, a big reduction in the total number of collected *Sergentomyia* (from 6927 in 2005 to 2673 for this current study) was observed. Particularly, we observed a significant reduction in the total number *S. darlingi*, found positive for *L. major* DNA in Mali [16], from 8 in 2005 [2] to 1 in the current study. Moreover, the density of sand flies per house per night in indoor collections by CDC light traps was reduced from 86.5 to 4.5 (for *Phlebotomus*), and 112.8 to 50.5 (for *Sergentomyia*), from 2005 [2] to the current study (see Additional file 1: Table S1). In June 2016, one month before the annual house spraying for mosquito control, a total of 105 *P. duboscqi* were caught in indoor collections by CDC light traps and aspiration. Due to the spraying undertaken in July, we compared current collections from March to June to historical collections [2]. We found a significant reduction in the density of *P. duboscqi* from 655 in 2005 [2] to 122 in the current study. In India, IRS/LLINs also led to significant reductions in the numbers of *P. argentipes* within sprayed houses for at least four weeks after the spraying [17]. Interestingly, the IRS/LLINs did not have an impact on the sand fly biting rate where a small proportion of *P. duboscqi* with blood meals was collected early after IRS/LLINs. The same observation has been demonstrated with malaria-vector human biting rates in IRS/LLINs sites by the President Malaria Initiative Programme [4]. Since the infection rate in wild-caught sand flies is usually low [18], microscopic evaluation on our study did not reveal any infected sand flies. This may have been due to the low number of dissected females (64 females) and to the low number of parasites in the inspected specimens. The incremental increase of the sand fly infection proportion from 2.7% in 2006 [2] to 3.5% in this study is probably due to the current analysis of individual female sand flies instead of pools as done previously [2].

Before IRS/LLINs, the incidence of *Leishmania* infection in humans was around 9.0% for the study area for two years, 2007 and 2008 [1]. After IRS/LLINs, our data indicate a large decrease in *Leishmania* exposure to 4.2% over the period 2008–2016 in these same villages. The same was observed in Bangladesh, India and Nepal, where IRS subsequently reduced the number of VL cases [12, 19]. Our *Leishmania* exposure data suggest that for Mali, the collateral benefit to the human population was clear since the transmission of *L. major* has been curbed. Additionally, in this study, we observed a big reduction in exposure to *Leishmania* in children under 10 years-old, and we theorize that this could be probably due to IRS/LLINs. Unfortunately, as most villages in the area were included in the campaign, we could not use a control village to link these findings directly to IRS/LLINs. Additionally, the conversion of individuals from a negative LST status in 2008 [1] to a positive LST during this study may be due to their exposure to *Leishmania* before IRS/LLINs started. Alternately, older subjects may have travelled to *Leishmania*-endemic areas that were not covered by the IRS/LLINs campaign.

Though this study demonstrated the effectiveness of IRS/LLINs in controlling sand fly populations, caution should be exercised in their long-term deployment to avoid the emergence of insecticide resistance in vector populations. Alternate methods, such as vaccine development, remain of paramount importance for the control of zoonotic cutaneous leishmaniasis due to *L. major* infections.

## Conclusions

Compared to historical data, the current study revealed the reduction in the density of sand flies following five years of IRS/ LLINs for malaria vector control. This study provides significant evidence that IRS/LLINs used in mosquito control in Mali has had a significant impact on the sand fly vector, in turn reducing the rate of exposure to *Leishmania major*.

## Additional files

Additional file 1: **Table S1.** Number of collected sand flies per household per month before and after IRS in the study area. Before IRS/LLINs, the number of collected flies per household ranged from 205 to 649 with a median of 293.00 and variance of 19778.94. After IRS/LLINs, the number of collected flies per household ranged from 25 to 159 with a median of 59.00 and variance of 3123.29. (DOCX 16 kb)

Additional file 2: **Table S2.** Meteorological information for the region of Segou, 2004 to 2017. (DOCX 19 kb)

## Abbreviations

CL: Cutaneous leishmaniasis
IRS: Indoor Residual Spraying
LLINs: Long-lasting insecticide-treated bednets
LST: Leishmanin skin test
VL: Visceral leishmaniasis
