# Supplementary material for: Impact of insecticide-treated bednets and indoor residual spraying in controlling populations of Phlebotomus duboscqi, the vector of Leishmania major in Central Mali
Source: Parasit Vectors. 2018 Jun 14;11:345. doi: 10.1186/s13071-018-2909-2 (PMC6000934; doi:10.1186/s13071-018-2909-2)
Supplement: Supplementary file 1 — Table S1. Number of collected sand flies per household per month before and after IRS in the study area. Before IRS/LLINs, the number of collected flies per household ranged from 205 to 649 with a median of 293.00 and variance of 19778.94. After IRS/LLINs, the number of collected flies per household ranged from 25 to 159 with a median of 59.00 and variance of 3123.29. (DOCX 16 kb) [file 13071_2018_2909_MOESM1_ESM.docx]

**Additional file 1: Table S1. Meteorological information for the region of Segou, 2004 to 2017.**

| **Years** | **Average temperature (°C)** | **Average rainfall (mm)** |
| --- | --- | --- |
| 2004 | 31.45 | 563.6 |
| **2005** | **28.8** | **555.9** |
| 2006 | 29.9 | 619.9 |
| 2007 | 30.3 | 521.2 |
| 2008 | 29.7 | 681.0 |
| 2009 | 29.5 | 737.0 |
| 2010 | 30.0 | 897.3 |
| 2011 | 29.6 | 693.2 |
| 2012 | 29.2 | 693.7 |
| 2013 | 29.3 | 547.9 |
| 2014 | 29.4 | 520.4 |
| 2015 | 29.3 | 599.0 |
| **2016** | **26.2** | **672.0** |
| 2017 | 26.1 | 567.0 |

Source: Meteorological Station; Segou region
