# Supplementary material for: Impact of insecticide-treated bednets and indoor residual spraying in controlling populations of Phlebotomus duboscqi, the vector of Leishmania major in Central Mali
Source: Parasit Vectors. 2018 Jun 14;11:345. doi: 10.1186/s13071-018-2909-2 (PMC6000934; doi:10.1186/s13071-018-2909-2)
Supplement: Supplementary file 2 — Table S2. Meteorological information for the region of Segou, 2004 to 2017. (DOCX 19 kb) [file 13071_2018_2909_MOESM2_ESM.docx]

**Additional file 2: Table S2:** **Number of collected sand flies in household before and after IRS in the study area**

| **Households** | **Before IRS** | | | | | | | | **After IRS** | | | | | | | |
| --- | --- | --- | --- | --- | --- | --- | --- | --- | --- | --- | --- | --- | --- | --- | --- | --- |
|  | March | April | May | June | July | August | September | Total | March | April | May | June | July | August | September | Total |
| K1 | 30 | 53 | 25 | 33 | 16 | 16 | 32 | **205** | 4 | 10 | 6 | 3 | 5 | 3 | 0 | **31** |
| K2 | 58 | 107 | 57 | 20 | 39 | 39 | 41 | **361** | 16 | 9 | 4 | 29 | 34 | 12 | 11 | **115** |
| K3 | 42 | 62 | 50 | 19 | 40 | 7 | 19 | **239** | 9 | 32 | 43 | 28 | 5 | 15 | 19 | **151** |
| K4 | 41 | 54 | 27 | 1 | 52 | 31 | 20 | **226** | 4 | 17 | 13 | 57 | 28 | 7 | 28 | **154** |
| K5 | 34 | 93 | 56 | 39 | 95 | 120 | 31 | **468** | 4 | 13 | 10 | 27 | 11 | 4 | 0 | **69** |
| S1 | 25 | 83 | 92 | 73 | 67 | 21 | 36 | **397** | 1 | 3 | 9 | 8 | 2 | 0 | 2 | **25** |
| S2 | 110 | 65 | 270 | 99 | 61 | 21 | 23 | **649** | 4 | 3 | 2 | 20 | 8 | 3 | 9 | **49** |
| S3 | 74 | 70 | 29 | 0 | 70 | 56 | 35 | **334** | 2 | 6 | 27 | 4 | 0 | 1 | 4 | **44** |
| S4 | 42 | 49 | 33 | 29 | 42 | 37 | 20 | **252** | 2 | 8 | 9 | 7 | 2 | 3 | 0 | **31** |
| S5 | 30 | 53 | 36 | 40 | 20 | 29 | 16 | **224** | 9 | 33 | 61 | 23 | 7 | 2 | 24 | **159** |
| **Total** | **486** | **689** | **675** | **353** | **502** | **377** | **273** | **3355** | **55** | **134** | **184** | **206** | **102** | **50** | **97** | **828** |
| *Mean* | *335.50* |  |  |  |  |  |  |  | *Mean* | *82.80* |  |  |  |  |  |  |
| *Median* | *293.00* |  |  |  |  |  |  |  | *Median* | *59.00* |  |  |  |  |  |  |
| *Minimum* | *205* |  |  |  |  |  |  |  | *Minimum* | *25.00* |  |  |  |  |  |  |
| *Maximum* | *649* |  |  |  |  |  |  |  | *Maximum* | *159.00* |  |  |  |  |  |  |
| *Variance* | *19778.94* |  |  |  |  |  |  |  | *Variance* | *3123.29* |  |  |  |  |  |  |
| *Std. Deviation* | *140.638* |  |  |  |  |  |  |  | *Std. Deviation* | *55.89* |  |  |  |  |  |  |
